# Assay of Phospholipase D Activity by an Amperometric Choline Oxidase Biosensor

**DOI:** 10.3390/s20051304

**Published:** 2020-02-27

**Authors:** Rosanna Ciriello, Antonio Guerrieri

**Affiliations:** Dipartimento di Scienze, Università degli Studi della Basilicata, Viale dell’Ateneo Lucano 10, 85100 Potenza, Italy; antonio.guerrieri@unibas.it

**Keywords:** choline biosensor, amperometric detection, overoxidized polypyrrole film, phospholipase D assay, phosphatidylcholine

## Abstract

A novel electrochemical method to assay phospholipase D (PLD) activity is proposed based on the employment of a choline biosensor realized by immobilizing choline oxidase through co-crosslinking on an overoxidized polypyrrole film previously deposited on a platinum electrode. To perform the assay, an aliquot of a PLD standard solution is typically added to borate buffer containing phosphatidylcholine at a certain concentration and the oxidation current of hydrogen peroxide is then measured at the rotating modified electrode by applying a detection potential of +0.7 V vs. SCE. Various experimental parameters influencing the assay were studied and optimized. The employment of 0.75% (v/v) Triton X-100, 0.2 mM calcium chloride, 5 mM phosphatidylcholine, and borate buffer at pH 8.0, ionic strength (I) 0.05 M allowed to achieve considerable current responses. In order to assure a controlled mass transport and, at the same time, high sensitivity, an electrode rotation rate of 200 rpm was selected. The proposed method showed a sensitivity of 24 (nA/s)⋅(IU/mL)^−1^, a wide linear range up to 0.33 IU/mL, fast response time and appreciable long-term stability. The limit of detection, evaluated from the linear calibration curve, was 0.005 IU/mL (S/N = 3). Finally, due to the presence of overoxidized polypyrrole film characterized by notable rejection properties towards electroactive compounds, a practical application to real sample analysis can be envisaged.

## 1. Introduction

Phospholipase D (PLD) is a lipolytic enzyme which in vivo hydrolyses the terminal phosphodiester bond of phospholipids, generating phosphatidic acid (PA) and a free polar head group [1]. In addition, in the presence of a primary alcohol, it is able to catalyze the in vitro transphosphatidylation of the naturally abundant phosphatidylcholine [2]. PLD has been isolated and sequenced from a variety of sources and, as recently reviewed [3], can be regarded as a key component of many cellular and physiological processes. 

In mammalian systems PLD is involved in cellular processes including membrane trafficking, cell proliferation, protein secretion, and metabolic regulation [1]. It has been associated with important pathological disorders such as thrombotic and neurodegenerative diseases, and several cancers [3]. Abnormalities in PLD activity in many human cancers have been noticed, highlighting a possible PLD role as a diagnostic biomarker and even as a target for drug discovery [4]. 

In plants, functional studies on PLD have shown its possible effects on membrane deterioration in response to stress injuries, senescence, aging, and pathogenesis [5]. 

In yeast, PLD activity has been shown to be involved mainly in sporulation and adaptation of nutrient utilization [6]. Bacterial PLD may act as virulence determinant and can mimic some reactions in cell [7]. Among hydrolytic enzymes from bacterial source, PLD from *Streptomyces Chromofuscus* is characterized by a notable transesterification capacity. This property can be exploited for the preparation of less abundant natural phospholipids, such as phosphatidylserine, phosphatidylglycerol, and novel artificial phospholipids of interest in the field of pharmaceutical and food industries, starting from crude or purified phosphatidylcholine [8,9]. Moreover, this bacterial PLD has been used as a model system for the mammalian enzyme since it mimics some reactions in cells [10]. A full understanding on the activation and inhibition mechanisms of this enzyme may be useful for in vivo studies on mammalian systems.

A wide variety of assay procedures have been realized for the determination of the activity of PLD enzymes. They can be substantially divided into two classes depending on the employment of exogenously-provided substrates, for in vitro measurements, or endogenous substrates for in vivo measurements [11]. In both cases the most used substrates are certainly radiolabeled phospholipids (radiolabeled acyl chains, phosphorus, or head group of the phospholipid). The radio-assay procedure has generally been reported to be fast and highly sensitive allowing to detect PLD in samples where its activity is very low, such as brain areas and peripheral tissues of mouse [12]. Despite these advantages, radio-assay is quite laborious and requires the employment of expensive and potentially health hazardous compounds. 

Other methods have been proposed based on titrimetric or pH-stat techniques [13], chromatographic technique [14], conductimetry [15], infrared spectroscopy [16] and nuclear magnetic resonance [17]. These methods, while being viable alternatives to radio-assay, are not suited for routine analysis. In this context, enzyme coupled assays have gain increasing importance. They are based on the quantification of choline released during the hydrolysis of phosphatidylcholine by PLD. The released choline is measured by the formation of a colored or fluorescent complex, upon the sequential reactions of choline oxidase and peroxidase enzymes [18].

In order to bypass the requirement of coupled enzymes, synthetic substrates of PLD have been used such as phosphatidyl p-nitrophenol [19]. A graphene-based nano-assembly has been proposed as a fluorescence biosensor for sensitive detection of PLD activity by employing a fluorescein-labeled phospholipid [20]: even if quite interesting, since miming a cellular membrane, this approach requires labeling, a fluorescence assay and was not applied to any real sample where endogenous substances could generate fluorescence signals interfering with the PLD assay. Nanoprobes for fluorescence detection and imaging of PLD in cell lysate were also realized based as well on rhodamine B-labeled phospholipids [21,22]. 

Alternative spectrophotometric methods were based on the determination of phosphatidic acid through iron (III) complexation [23] or through Ca^2+^ complexation with 8-hydroxyquinoline [24]. In this last case, a direct and continuous PLD assay was achieved exploiting the chelation-enhanced fluorescence property of 8-hydroxyquinoline.

The aim of the present work was to realize an assay method sensitive, although nonradioactive, and easy to realize, without requiring the use of synthetic phospholipids, derivatization reaction, and instrumentations particularly expensive and difficult to handle. We developed an enzyme coupled procedure, based on a choline amperometric biosensor, able to provide real time measurements and suitable for high throughput assays. Indeed, electrochemical biosensors are clearly advantageous since they can overcome some limitations of the spectroscopic methods: they require a minimum sample pretreatment and can be used also for the analysis of turbid or colored samples.

The employment of choline amperometric biosensors for PLD assay is almost unexplored. Vrbova et al. [25] reported a PLD assay method based on a Clark-type electrode modified by a nylon net co-immobilizing choline oxidase and catalase. Besides the limitations typical of oxygen sensors, another drawback was surely the immobilization technique adopted which was quite complex and time consuming, requiring an incubation period of the enzymatic solution of 4 days. 

The device we propose is surely easier to realize while assuring high enzyme stability. The approach we adopted is based on the detection of hydrogen peroxide which requires high applied potential thus causing severe interference problems from electroactive endogenous compounds presents in real samples. The modification of the Pt electrode by overoxidized polypyrrole, before choline oxidase (ChO) immobilization by co-crosslinking, could successfully solve this problem. This film is characterized by notable rejection properties towards interferents, as already demonstrated in the case of other enzymes and analytes [26,27,28]. 

The resulting device could be used in general to assay other enzymes than PLD which release choline as well. Indeed, we have already employed this biosensor to assay cholinesterase activity in human serum samples [29]. Having demonstrated the validity of such a sensor, in this work we wanted to extent its employment to the analysis of PLD by substantially modifying the reaction environment. Particularly, being PLD activity dependent on whether the substrate is readily accessible and on the presence of divalent cations at a certain concentration [3], a high sensor response was assured only upon having opportunely optimized the experimental conditions. As it will be shown, notable performances were achieved.

## 2. Materials and Methods

### 2.1. Materials

Choline oxidase (EC 1.1.3.17 from Alcaligenes species, 12 U/mg of solid), serum bovine albumin (fraction V), choline chloride, glutaraldehyde (grade II, 25% aqueous solution), L-α-phosphatidylcholine (Type XI-E: from fresh egg yolk, approx. 99%, 100 mg/mL solution in chloroform) and Triton X-100 were obtained from Sigma (Sigma Chemical Co.; St. Louis, MO, USA). Phospholipase D (EC 3.1.4.4., from *Streptomyces Chromofuscus*, 50,000 U/mL) was purchased from Biomol Research Laboratories Inc. (Butler Pike, Plymouth Meeting, PA, USA). Pig lung comes from a local butcher shop.

The above chemicals were of analytical reagent grade and were used without further purification with the exception of pyrrole (Aldrich, Steinheim, Germany) which was purified by distillation under vacuum by fixing the temperature at 62 °C. Choline chloride before its use was left to dry in a vacuum desiccator for 3 days in the presence of P2O5. 

Choline stock solutions were prepared in double distilled-deionized water and stored in the dark at 4 °C. Fresh L-α-phosphatidylcholine standard solutions were prepared daily by evaporating chloroform and then dissolving the required amounts in 50 mM borate buffer (pH 8.0) containing 0.75% (v/v) Triton X-100 and 0.2 mM calcium chloride. Phospholipase D solutions were prepared in borate buffer pH 8.0 unless otherwise stated. The PLD activity is expressed in International Units (IU) (1 IU releases 1 µmol of choline per minute) throughout the work.

### 2.2. Spectrophotometric Assay

Before use, PLD activity was assayed by an established colorimetric method using a reagent kit available from Biomol (Phospholipase D Assay Catalog SE-301). The assay comprises three reaction steps. In the first step, PLD catalyzes the hydrolysis of phosphatidylcholine to choline and phosphatidic acid. In the second step, the oxidation of choline catalyzed by choline oxidase produces two hydrogen peroxides. Peroxidase catalyzes the third step, in which the two hydrogen peroxides, phenol and 4-aminoantipyrine react to produce a quinonimine dye, with a millimolar extinction coefficient of 12.2 mM^−1^ cm^−1^ at 500 nm. Measurement of the absorbance at 500 nm can then be used to calculate the amount of choline produced by the phospholipase reaction.

### 2.3. Apparatus

To perform electrochemical experiments, an EG&G (Princeton Applied Research, Princeton, NJ, USA) model 263 A potentiostat/galvanostat was used. Data acquisition was accomplished through a M270 electrochemical research software (EG&G) version 4.23.

A conventional three electrode cell was used consisting of a platinum counter electrode and a saturated calomel reference electrode (SCE). The working electrode was a platinum disk (2 mm diameter) embedded in a poly(tetrafluoroethylene) (PTFE) body. Experiments under controlled mass transport were performed by a CTV101 Speed Control Unit, EDI 101 Rotating Disc Electrode (RDE) (Radiometer Copenhagen). A rotation rate of 200 rpm was typically adopted unless otherwise stated.

Spectrophotometric measurements were performed using a single beam LKB Biochrom Ultrospec II 4050 UV/Visible spectrophotometer (Biochrom Ltd., Cambridge, UK). 

### 2.4. Biosensor Preparation

In order to assure a reproducible electrode surface, before each modification Pt substrates were subjected to a cleaning procedure comprising a first step of dipping in hot nitric acid for few minutes. Then the electrode was polished by mechanical abrasion employing an alumina slurry with particles of 0.05 µm. Finally, to remove any adsorbed alumina, the electrode was copiously rinsed with bi-distilled water and sonicated for several minutes.

Polypyrrole films (PPy) were electrosynthesized potentiostatically using a 0.4 M pyrrole solution in 0.1 M KCl electrolyte. A selected potential of +0.7 V versus SCE was applied and polymer growth was stopped when a deposition charge of 300 mC/cm^2^ was passed. The formed film was then immersed in a phosphate buffer (pH = 7.0, I = 0.1 M) and overoxidized by applying a fixed potential of +0.7 V versus SCE. After a time of at least 7 h, a steady-state background current was reached and the overoxidation process was stopped. The resulting modified electrode (Pt/PPyox) was rinsed with bi-distilled water and air-dried at room temperature before enzyme immobilization.

Choline oxidase immobilization was carried out by following a procedure already reported [30]: 16 mg of bovine serum albumin (BSA) and 1 mg of ChO were dissolved in 300 µL of a phosphate buffer solution (I = 0.1 M, pH = 6.5); 30 µL of 2.5% glutaraldehyde solution (25% glutaraldehyde solution diluted 1:10 with phosphate buffer) were then added; 3 µl of the resulting solution were pipetted onto the Pt/PPyox working electrode surface and spread out to completely cover the electrode surface avoiding air bubble formation. Finally, the modified electrode was air-dried at room temperature and soaked in the background electrolyte to remove weakly bound or adsorbed enzyme. The enzyme electrode was stored in phosphate buffer, pH 6.5, I 0.1 M, at 4 °C in the dark.

### 2.5. Electrochemical Measurement

A detection potential of +0.7 V versus SCE was adopted to perform all the electrochemical measurements. PLD assay was performed through batch addition experiments: the modified electrode was immersed in a borate buffer (I = 0.05 M, pH = 8.0) solution stirred at 200 rpm and containing calcium chloride 0.2 mM and Triton X-100 0.75% (v/v); an aliquot of a phosphatidylcholine solution, and then an aliquot of a standard PLD solution were added into the cell and the current variation with time was recorded. A temperature of 37 °C was maintained for the duration of the assay by a thermostatic bath.

### 2.6. Real Sample Analysis

Extraction of PLD from pig lung was performed accordingly to a procedure described elsewhere [31]. Briefly, about 500 g of pig lung was minced and homogenized in sucrose buffer at 4 °C, centrifuged and stored at −80 °C until its use. Just before analysis, the thawed homogenized was diluted in buffer to reach the desired PLD activity, stirred, further centrifuged, and the desired supernatant aliquot was added to the electrochemical cell for PLD assay. 

## 3. Results and Discussion

**Scheme 1:**
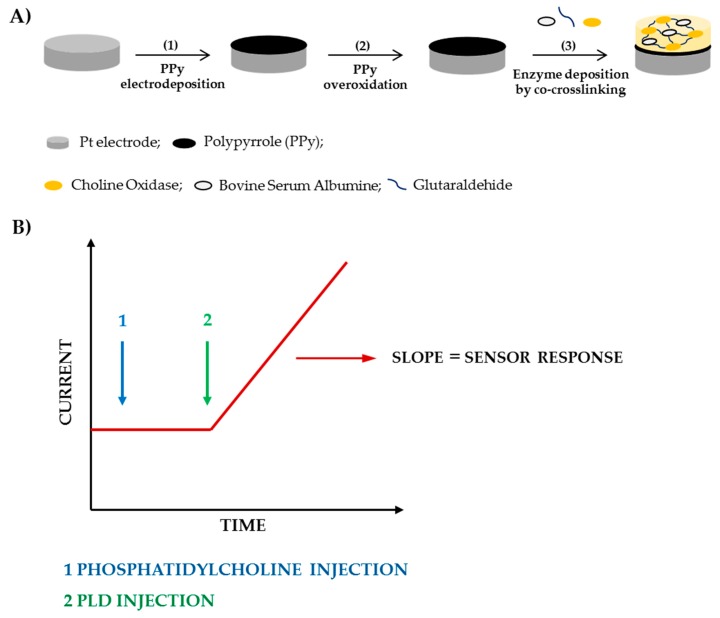
Fabrication steps of the biosensor (**A**) and current signal acquisition (**B**).

In the present work, we have proposed an assay method based on the employment of a modified Pt/PPyox/ChO electrode, realized following the fabrication steps shown in Scheme 1, which is immersed in a thermostated solution of phosphatidylcholine and kept under a fixed rotation rate. Then, to perform the assay, an aliquot of a standard PLD solution is injected in the cell which causes the release of free choline (Ch). The immobilized ChO catalyzes the oxidation of Ch to betaine with the subsequent production of hydrogen peroxide. At the electrode surface, opportunely polarized, hydrogen peroxide undergoes progressive oxidation producing a current signal which increases linearly with time. PLD activity in the cell solution can be evaluated from the slope of the current signal increase, as shown in Scheme 1, after having opportunely calibrated the sensor.

Following are the reactions involved to produce the sensor response

(1) $$\mathrm{phosphatidylcholine}\;+\;2H_{2}O\;\overset{PLD}{\to }\;\mathrm{phosphatidic}\;\mathrm{acid}\;+\;\mathrm{choline}\;$$

(2) $$\mathrm{choline}\;+\;2O_{2}\;+\;H_{2}O\;\overset{ChO}{\to }\;\mathrm{betaine}\;+\;2H_{2}O_{2}$$

H_2_O_2_ → O_2_ + 2H^+^ + 2e^-^ (3)

The notable performances of the choline oxidase biosensor towards choline detection have already been summarized in our previous work [29]. Fast response time, high sensitivity, wide linear range, and significant long-term stability were achieved. Furthermore, the presence of the underlying overoxidized polypyrrole film guaranteed a notable rejection of interferent compounds, electroactive at the high overpotential required to detect H_2_O_2_, thus allowing the application of the sensor to assay Cholinesterase in real samples as complex as human serum [29]. Electrochemical, XPS, and ESEM characterizations of these biosensors were already described elsewhere [28,29,30,32,33,34,35]. Based on these important achievements, in the present work we wanted to employ such a biosensor to develop a novel PLD assay method.

PLDs from the culture broth of *Streptomyces* are commercially available or can be prepared by fermentation from some *Actinomycetes* strains [36,37]. Imamura and Horiuti provided information on the purification of PLD from Streptomyces Chromofuscus secreted into the growth medium [38]. They partially characterized this bacterial PLD furnishing a molecular weight of 57 kDa and an isoelectric point of pH 5.1. To optimize the enzymatic reaction, alkaline pH values are required in the range 8–9 and the presence of detergent (Triton X-100, deoxycholate), Ca^2+^ or both of them revealed able to significantly increase the velocity [38]. Indeed, like the eukaryotic enzymes PLD from Streptomyces Chromofuscus requires Ca^2+^ ion for activity whereas, unlike other phospholipases, does not exhibit “interfacial activation” or “surface dilution” [39,40]. This means that this enzyme does not show preference for substrate in the form of micellar short chain phospholipids rather than monomeric ones [39] and its specific activity does not depend on the mole fraction of substrate in a micelle or bilayer surface [40]. Nevertheless, considering that the catalytic reaction involves vesicle substrates, the transfer of PLD from aqueous solution to the lipid-water interface is a required step for the reaction to take place. 

The binding mechanism that has been proposed for the enzyme is a two-step ping-pong like ordered one: the substrate combines with the enzyme giving a covalent phosphatidyl-enzyme moiety with release of choline; then, water attacking to the covalent adduct induces the release of phosphatidic acid [41]. The cleavage of the P-O bond is caused by the nucleophilic attack of water on the distal phosphate ester bond. It is worth noting that, in the presence of a primary alcohol at a high concentration, the phosphatidyl-enzyme intermediate can be decomposed to a different phospholipid.

It is evident that PLD activity is affected by various experimental variables, such as substrate accessibility, presence of a bication at a proper concentration, and a surfactant able to solubilize at the right extent the substrate, which need a careful investigation in addition to the more usual pH and composition of the reaction medium. An optimization study of the reaction conditions has therefore been carried out with the aim to implement a method able to quantify low PLD activities in a reproducible way and without any interference.

### 3.1. Influence of Surfactant 

Phospholipases are generally more active on liposomes, mixed micellar deriving from the dispersion of phospholipids substrate in detergents, or phospholipids solubilized in organic solvents [42]. Particularly, PLD from *Streptomyces Chromofuscus* is reported to exhibit higher activity towards monomers and mixed micelles than substrate present in lipid vesicles [43]. When assaying PLD activity it is then a common practice to employ mixtures of phospholipid and detergents. PLDs from different sources are activated by anionic or neutral detergents [44] whose influence on the enzymatic activity has been attributed mainly to the micellization of phospholipids substrates. Changes of the physicochemical properties of the phospholipid bilayers are not to be excluded. Practically, detergents exert their action by destroying liposome structure favoring the gradual formation of mixed micelles. Indeed, the formation of mixed micelles is the final goal achieved during the interaction of detergents with liposome. At the beginning of such an interaction, surfactant monomers are incorporated within the lipid bilayer and, as a consequence, vesicle dimensions increase. Then, as the surfactant concentration is increased, phospholipids solubilization gradually occurs and liposomes along with mixed micelles are both presents. Finally, only mixed micelles are present as phospholipids are completely solubilized. 

Triton X-100 is one of the most common surfactants employed for PLD assay. It is a non-ionic detergent with an average length of approximately 9.5 oxyethylene units per detergent molecule. Its wide employment can be justified considering that it is a mild non-denaturing detergent: even at concentrations up to 0.5% it usually does not damage most enzymes. For lysing cells lower concentrations of about 0.1% are sufficient. 

**Figure 1:**
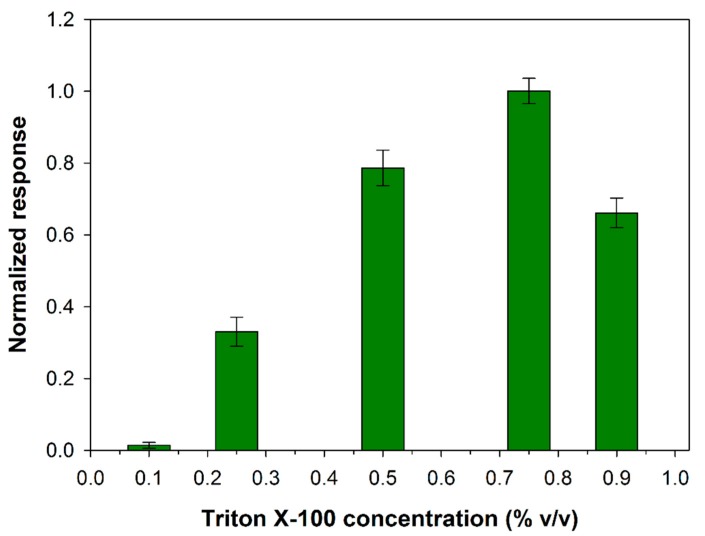
Normalized response (i.e., response/maximum response) of a typical Pt/PPyox/ChO biosensor to PLD 0.22 IU/mL as a function of Triton X-100 concentration. Supporting electrolyte: Tris buffer pH 8.0, I 0.05 M containing 10 mM calcium chloride, and 5 mM phosphatydylcholine. Electrode rotation rate: 1000 rpm.

In the present work, the appropriate Triton X-100 concentration to employ for PLD assay has been evaluated. At this aim experiments have been carried out by varying the detergent concentration in the range 0.1–0.9% (v/v), while fixing the other experimental conditions close to the values adopted in the spectrophotometric assay (phosphatidylcholine 5 mM, PLD 0.22 IU/mL, calcium chloride 10 mM, Tris buffer pH 8.0, I 0.05 M). In Figure 1 the variation of the slopes computed from the current signals increments is illustrated as a function of the detergent concentration. As it can be seen, in the first part of the graph biosensor response increases as detergent concentration is increased in agreement with a more efficient phosphatidylcholine dissolution at higher contents of Triton X-100. The graph shows a maximum located at a concentration of about 0.75%. A further increase in detergent concentration probably causes a partial enzyme denaturation thus justifying the response decrease evidenced in the second part of the graph. 

Indeed, many enzymes remain active in the presence of Triton X-100 even at concentration of 1%, as it was reported for ChO [45]. As concern the effect of detergent on the activity of PLD, it was showed that when the activity was maximally increased by Ca^2+^ (10 mM) detergent was inhibitory at lower concentrations (completely at about 0.8 mM) and stimulating at higher concentrations (maximally at 4.5 mM) [38]. At the experimental conditions we adopted an inhibition effect on PLD from detergent is to be excluded. On the other hand, it was reported that the enzymatic reaction rate is optimal at a certain detergent to phospholipid ratio, a further increase in the molar ratio of Triton X-100 to phospholipid lead to progressive decrease in the enzymatic activities probably due to a sort of dilution of the micellar surface concentration of phosphatidylcholine [38]. In conclusion, the effect of the detergent on Phospholipase D activity would seem to be related essentially to the formation of mixed micelles that in the present experimental conditions is favored at a Triton concentration of 0.75%. This value was adopted from now on to assay PLD activity.

### 3.2. Influence of Calcium Concentration 

**Figure 2:**
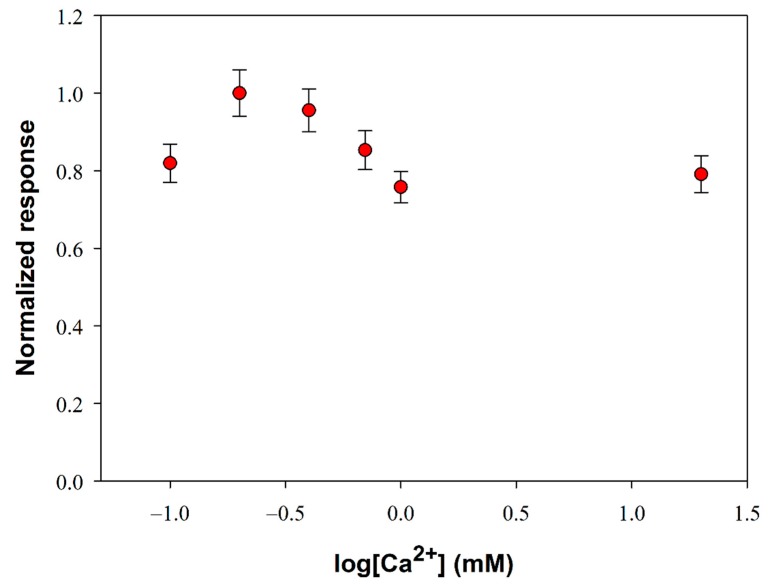
Normalized response (i.e., response/maximum response) of a typical Pt/PPyox/ChO biosensor to PLD 0.22 IU/mL as a function of Ca^2+^ concentration. Supporting electrolyte: Tris buffer pH 8.0, I 0.05 M containing 0.75% (v/v) Triton X-100 and 5 mM phosphatidylcholine. Electrode rotation rate: 1000 rpm. Each value represents the mean of triplicate measurements; the error bars represent the relevant standard deviations.

The PLD from *S. Chromofuscus* is a water-soluble enzyme unlike its natural substrate which forms macromolecular associations. Since the PLD activity takes place in a heterogeneous medium, factors that could enhance enzyme removal from the soluble part of the medium (i.e. aqueous solution) to the particulate part (i.e. the lipid vesicles or monolayer) are of relevance. At this aim, ultrafiltration experiments showed that Ca^2+^ at micromolar concentration levels was essential for the PLD binding to PC vesicles suggesting that the binding of Ca^2+^ to PLD is followed by a conformational change of PLD and then the enzyme likely associates with PC vesicles [46]. Besides favoring the enzyme location at the water–lipid interface, calcium ion is reported to increase also the catalytic activity of PLD: it plays a key role in the phosphatidyl enzyme geometry for the nucleophilic attack of water on the P-O bond [47]. Considering then the PLD requirement for calcium, experiments were performed to determine the extent to which added Ca^2+^ affects its activity. Figure 2 shows the influence of calcium content on the biosensor response at fixed phosphatidylcholine and PLD concentrations. As it is possible to see, PLD activity increases as calcium concentration is increased up to a maximum at about 0.2 mM (log[Ca^2+^] = −0.7) and then decreases to reach a quite constant value from calcium concentrations higher than 1 mM. 

It is worth noting that, despite the small variations, an ANOVA analysis of data points (F-value 10.6, *p*-value 0.0004373 at a confidence level of 95%) evidenced that there’s a significant difference between the mean response values of Figure 2. A more detailed treatment of the ANOVA analysis is reported in the Supplementary Materials. A calcium concentration value of 0.2 mM was therefore employed in the following experiments to optimize PLD assay. 

### 3.3. Influence of Electrode Rotation Rate 

**Figure 3:**
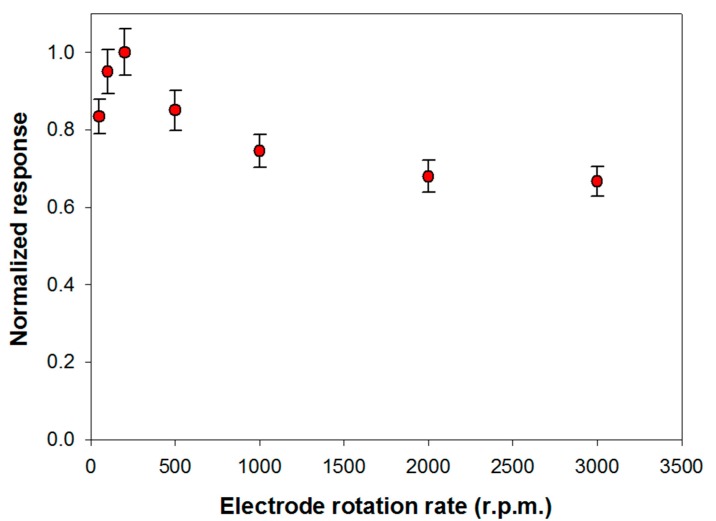
Normalized response (i.e., response/maximum response) of a typical Pt/PPyox/ChO biosensor to PLD 0.22 IU/mL as a function of electrode rotation rate. Supporting electrolyte: Tris buffer pH 8.0, I 0.05 M containing 0.75% (v/v) Triton X-100, 0.2 mM calcium chloride, and 5 mM phosphatidylcholine. Each value represents the mean of triplicate measurements; the error bars represent the relevant standard deviations.

The influence of substrate mass transport on the analytical performances of the biosensor was investigated. This study has been carried out in Tris-HCl buffer (pH 8.0, I 0.05 M) containing Triton X-100 and Ca^2+^ at the optimized concentrations respectively of 0.75% (v/v) and 0.2 mM. Under these experimental conditions, the variation of the slope of the signal current upon the enzymatic conversion of phosphatidylcholine 5 mM from PLD 0.22 IU/mL has been measured, by varying the rotation rate of the electrode from 50 to 3000 rpm. The upper limit of the explored range was limited by the well-known tendency of detergents to skim. Figure 3 shows the signal slopes variation as a function of the rotation rate at a Pt/PPyox/ChO biosensor. As expected in the case of a process limited by the mass transport in solution, the profile acquired shows in the first part an increment of the response as rotation rate is increased up to about 200 rpm. At higher rotation rates the slope of the signal decreases as rotation rate is increased, thus suggesting that the system is passing from an external diffusive control to an enzymatic kinetic control [48].

Particularly, considering that solution stirring promotes the interaction PLD/phosphatidylcholine, the homogeneous catalytic process is unlikely to constitute the rate determining step. The whole bienzymatic process is reasonably controlled by the kinetic of the enzymatic reaction catalyzed by choline oxidase which occurs at the modified electrode. Indeed, the profile at higher rotation rates showed in Figure 3 is similar to that, here not reported, observed when using choline oxidase biosensor for choline sensing. On the other hand, hypothesizing the PLD catalysis in solution as the rate determining step, current signal would have been independent from the rotation rate. This behavior has already been noticed when the same choline biosensor was employed to assay ChE activity in solution following a similar reactions scheme [29].

In conclusion, to assure an appreciable sensitivity under controlled mass transport, a rotation rate of 200 rpm has been adopted in the following experiments.

### 3.4. Influence of pH 

Being the proposed method based on the employment of an auxiliary enzyme, i.e., choline oxidase, before optimizing the pH for PLD assay, the effect of the buffer composition on the response of choline oxidase biosensor was examined using phosphate, glycine, tris, and borate buffers. The results, reported in Figure S2 of Supplementary Materials, showed that phosphate and borate buffer allowed to achieve the highest signals. One of the most employed biological buffers is surely Tris which however does not always assure the best analytical results [49]. As a confirmation, in the present study its employment resulted in the lowest response among all the studied buffers. Campanella et al. indeed evidenced a partial inhibition of choline oxidase in Tris buffer [50].

**Figure 4:**
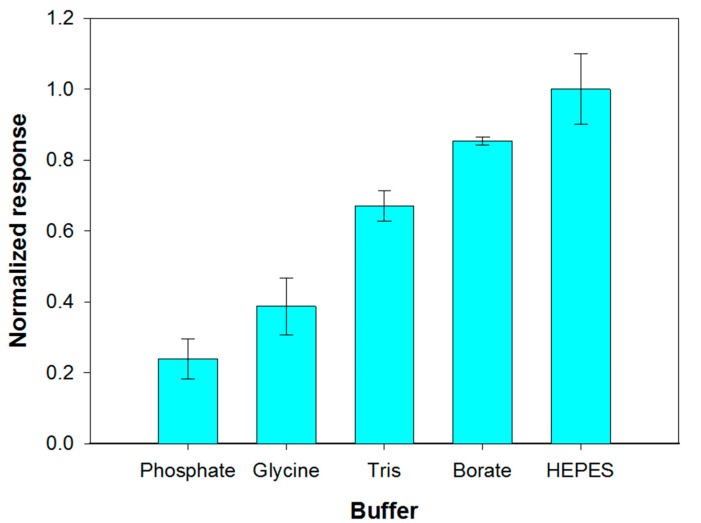
Effect of buffer composition (pH 8.0, I 0.05 M) on choline biosensor response to PLD 0.22 IU/mL. Triton X-100 concentration: 0.75% (v/v). Calcium chloride concentration: 0.2 mM. Phosphatidylcholine concentration: 5 mM. Electrode rotation rate: 200 rpm. Each value represents the mean of triplicate measurements; the error bars represent the relevant standard deviations.

The reaction environment in which the enzymatic hydrolysis of phospholipids containing choline is carried out is usually composed of 50 mM Tris buffer at pH 8.0, calcium chloride and Triton X-100 [51]. Considering that the response of the choline biosensor was dramatically reduced in Tris buffer, as it was previously showed, the possibility of carrying out PLD assay in alternative buffers has been evaluated. At this aim phosphate, glycine, Tris, HEPES and borate buffers (0.05 M, pH 8.0) containing 0.75% (v/v) Triton X- 100- and 0.2-mM calcium chloride were employed. With the aim to discriminate which buffer allowed high sensitivity while assuring appreciable reproducibility, triplicate assay experiments have been carried out for each investigated system. The mean values of the current slopes with the relevant standard deviations are shown in Figure 4.

The best results in term of sensitivity were obtained by employing HEPES and borate buffer. Nevertheless, HEPES buffer provided a poor repeatability of the analytical signal which progressively decreased after successive measurements, as it was already evidenced by Marazuela et al. [45]. Phosphate buffer showed poor reproducibility and sensitivity attributable to precipitation processes at the micromolar calcium concentrations required to maximize PLD activity. Such a buffer was therefore not used even if its employment assured the best performances for choline biosensor (Figure S2). Borate buffer, allowing the best response in terms of both precision (relative standard deviation of 1.3% (n = 3) on intraday measurements) and sensitivity was therefore employed for all further experiments.

**Figure 5:**
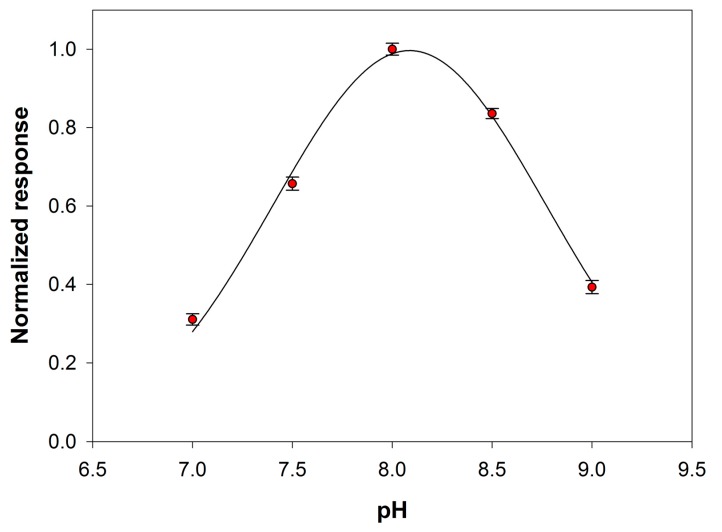
Normalized response (i.e., response/maximum response) of a typical Pt/PPyox/ChO biosensor to PLD 0.22 IU/mL as a function of pH. Supporting electrolyte: borate buffer pH 8.0, I 0.05 M containing 0.75% (v/v) Triton X-100, 0.2 mM calcium chloride, and 5 mM phosphatidylcholine. Electrode rotation rate: 200 rpm.

The effect of the pH solution on the analytical signal was then tested within the range 7–9. At this aim the behavior of both the primary enzyme and the auxiliary enzyme upon varying pH was considered. Preliminary experiments, reported in Figure S3 of Supplementary Material, showed that the maximum sensitivity towards choline of the device Pt/PPyox/ChO falls in the pH range 8.5–9.5. PLD is reported to show in solution a maximum activity at a pH close to 8.0 [38]. Figure 5 shows the signal slopes variation as a function of pH at a Pt/PPyox/ChO biosensor upon the addition of phosphatidylcholine (5 mM) and PLD (0.22 IU/mL). The curve evidences a maximum located at pH of about 8.0 and therefore in good agreement with the value reported in literature. Subsequent studies have been therefore carried out at pH 8.0 since at this value the activities of both PLD and ChO are almost at maximum.

### 3.5. Optimization of Substrate Concentration

PLD from *Streptomyces Chromofuscus*, like bacterial PLDs in general, has broader substrate specificity than the eukaryotic enzymes. Such an enzyme is able to hydrolyze phosphatidylserine, phosphatidylethanolamine, phosphatidylglycerol, lysophosphatidylcholine, and sphingomyelin. Phosphatidylcholine is considered however the best substrate for hydrolysis. In the present work, L-α-phosphatidylcholine from egg yolk was employed with a mean molecular weight of about 768 Da. It is worth noting that complications arise when drawing kinetic data on phospholipases. These enzymes, indeed, acting on water-insoluble substrates, display an enhanced activity when using substrates organized in aggregates. This feature inevitably determines a dependence of the kinetic data on the particular phase in which phospholipids are organized in the reaction mixture. For this reason, we have experimentally verified at which substrate concentration, and then organization, PLD saturation was guaranteed at the experimental conditions already optimized. A calibration graph has been then constructed by varying the phosphatidylcholine concentration realized in the electrolyte solution (borate buffer pH 8.0, I 0.05 M containing 0.75% Triton X-100, and 0.2 mM Ca^2+^) containing PLD at a concentration of 0.22 IU/mL, and holding the electrode under rotation at a speed of 200 rpm.

**Figure 6:**
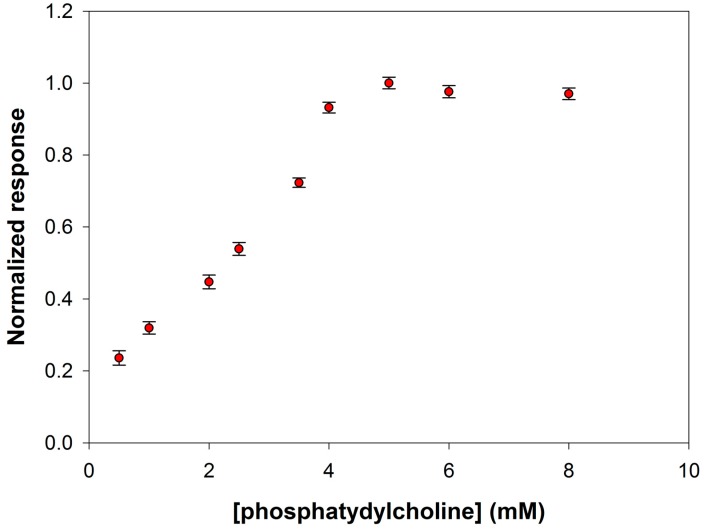
Normalized response (i.e., response/maximum response) of a typical Pt/PPyox/ChO biosensor to PLD 0.22 IU/mL as a function of phosphatidylcholine concentration. Supporting electrolyte: borate buffer pH 8.0, I 0.05 M containing 0.75% (v/v) Triton X-100, and 0.2 mM calcium chloride. Electrode rotation rate: 200 rpm.

Figure 6 shows the behavior of the signal current slopes at substrate concentration values ranging from 0.5 to 8 mM. As it is possible to see, a phosphatidylcholine concentration of 5 mM assured substrate saturation condition for PLD and was therefore adopted to realize the calibration curve for enzyme assay.

### 3.6. Calibration Graphs for PLD Assay

**Figure 7:**
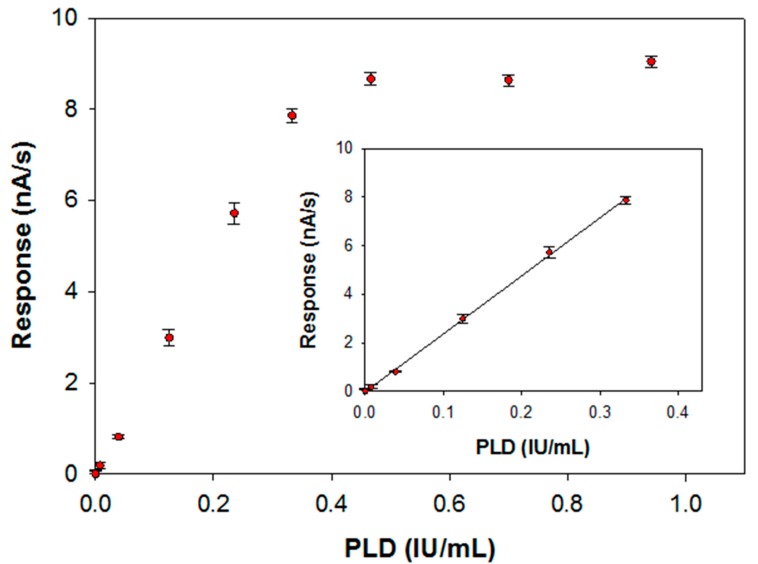
Calibration curve for phosphatidylcholine-PLD system carried out at a typical Pt/PPyox/ChO biosensor. Supporting electrolyte: borate buffer pH 8.0, I 0.05 M containing 0.75% (v/v) Triton X-100, 0.2 mM calcium chloride and 5 mM phosphatidylcholine. Electrode rotation rate: 200 rpm. Inset: linear interval of the calibration curve.

The calibration curve for PLD assay has been derived by injecting in the electrochemical cell the substrate at the concentration value previously optimized and then by introducing an aliquot of an enzyme standard solution at a fixed temperature of 37 °C. The response has been evaluated, as usual, by the slope of the current signal acquired at the modified electrode. In Figure 7 the calibration graph obtained over all the PLD activities investigated is reported whereas in the inset the linear range of the calibration curve is also illustrated. Fitting of the linear part of the calibration graph gave a regression line with a slope of 23.9 ± 0.2 (nA/s)·(IU/mL)^−1^ and an intercept of (−0.0148 ± 0.0382) nA/s (correlation coefficient better than 0.999). Due to the significant accumulation of the enzymatic products during the observation times, a deviation from linearity is observed from an enzyme activity of 0.33 IU/mL. The within-day coefficients of variation for replicate (n = 5) PLD assays were 4.15% and 5.18% at 0.22 and 0.04 IU/mL PLD levels, respectively; the between-days (n = 6) coefficient of variation was 9.4% at 0.14 IU/mL PLD levels. The operational stability of the assay was tested doing replicate PLD assays (at 0.22 IU/mL PLD level) during a day (n = 10): the relevant variation was comparable (confidence level of 95%) with the within-day coefficients of variation so the biosensor response could be considered almost stable. Finally, the long-term stability was proved by evaluating the sensitivity of several calibration curves (n = 15) for PLD assay (at 0.22 IU/mL PLD level) during an extended period (biosensor stored in buffer at 4 °C at the dark when not used): after 30 days, the sensitivity dropped to about 25% of its initial value. 

The limit of detection estimated from the linear regression equation was 0.005 IU/mL, corresponding to an absolute amount of 0.03 IU (30 nmol of choline per minute). This amount is suitable for practical determination of PLD in real samples. As an example, in the clinical field PLD activity in normal mammary cell attests to levels detectable with our device. In mammary carcinoma cell lysate PLD activity was found to be considerable higher with respect to normal cell (2921.4 ± 216.7 U/L vs. 408.6 ± 28.7 U/L) [21]. 

A direct comparison with existing PLD assay methods is complicated by the different modalities in which enzyme activity is expressed. Chemiluminescence assays have been reported in which liberation of just 75 pmol of choline per minute was detectable [52]. Method precision was however affected by the presence of substances which react with hydrogen peroxide and proteins at concentrations above 0.2% (w/v) which can cause quenching phenomena of the luminol reaction therein employed. Fluorometric detection and radiometric assay are surely highly sensitive techniques [23,24] but need synthetically modified fluorogenic substrates or special equipment to be performed. As concerns the electrochemical method previously described to assay PLD, based on the employment of a choline amperometric biosensor [25], a limit of detection for choline of 0.05 mM was reported and then substantially higher with respect to the value of 0.1 µM at pH 8 that we evaluated in our previous work [29]. Moreover, as it was already stressed, the biosensor realization was quite laborious and time consuming [25]. Compared to existing analytical assays, the present method is therefore simple and easy to operate, representing a valid alternative in ascertaining PLD activity in various fields of interest.

Preliminary experiments concerning the application of the proposed assay in real samples focused on the determination of PLD activity on crude extracts from pig lung. Lungs were chosen either for its high PLD activity [53] either because its crude extract is surely a complex biological matrix containing endogenous substances (e.g., ascorbate) which can interfere with the present electrochemical assay. Yet, the notable ability of PPyox polymer to reject interfering and fouling species in electrochemical analysis ensured proper measurements of PLD activity even in complex matrixes like this. In fact, analysis of pig lung crude extracts, after proper dilution, gave a specific activity of 1.50 ± 0.08 mIU/mg which agree with that obtained by the standard colorimetric method (see Materials and Methods section) used here for PLD assay (values from both assays not significantly different at a 95% confidence level); more importantly, the assay of pig lung crude extracts gave specificity activity values in agreement with those already reported elsewhere [31]. 

## 4. Conclusions

The novel electrochemical procedure for PLD assay proposed in this work is based on the employment of a bilayer choline biosensor composed of a co-crosslinked choline oxidase coating deposited on a permselective overoxidized polypyrrole film electrosynthesized on a platinum electrode. The optimization of the experimental parameters affecting PLD activity (e.g., pH and composition of the supporting electrolyte, hydrodynamic conditions, calcium chloride and surfactant concentration) allowed to obtain calibration curves characterized by appreciable sensitivity, wide linear range and adequately low limit of detection. Furthermore, the electrosynthesis of a polymer film with built-in permselectivity could realize a biosensor suitable for real matrices analysis being interferences from endogenous compound, electroactive at the applied potential, minimized. In conclusion, our device proved as an effective and novel method for the determination of PLD activity. Further experiments concerning its application to assay such an enzyme in biological samples are under way in our laboratory.

## Supplementary Materials

The following are available online at https://www.mdpi.com/1424-8220/20/5/1304/s1, Section S1: Influence of calcium concentration: ANOVA analysis. Figure S1: Normalized response of a typical Pt/PPyox/ChO biosensor to PLD 0.22 IU/mL as a function of Ca^2+^ concentration. Experimental conditions as in Figure 2. Different labels indicate significantly different values. Figure S1: Dependence of the choline biosensor sensitivity on the buffer composition (pH 8.5, I 0.1 M). Electrode rotation rate: 1000 rpm, Figure S2: Influence of pH on the response of a typical Pt/PPyox/ChO biosensor. Supporting electrolyte: acetate/phosphate/borate buffer I 0.1 M. Electrode rotation rate: 1000 rpm.
