# Supplementary material for: Assay of Phospholipase D Activity by an Amperometric Choline Oxidase Biosensor
Source: Sensors (Basel). 2020 Feb 27;20(5):1304. doi: 10.3390/s20051304 (PMC7085753; doi:10.3390/s20051304)
Supplement: Supplementary file 1 [file sensors-20-01304-s001.zip › sensors-709855-supplementary.doc]

Assay of Phospholipase D activity by an amperometric choline oxidase biosensor

Rosanna Ciriello* and Antonio Guerrieri

Dipartimento di Scienze, Università degli Studi della Basilicata, Viale dell’Ateneo Lucano 10, 85100 Potenza, Italy

***** Correspondence: rosanna.ciriello@unibas.it; Tel.: +39-0971-205944

S1. Influence of calcium concentration: ANOVA analysis

An ANOVA test was performed on the experimental data points illustrated in Figure 2 showing the influence of calcium concentration on the biosensor response.

Results of the ANOVA test performed on experimental data are listed below:

F value	Pr(>F)

log[Ca^2+^]	10.6	0.0004373 ***

The F-value is moderately high and the p-value is smaller than 0.05 (95% confidence level). So, we can conclude there’s a significant difference between the means.

Moreover, results of multiple mean comparison are listed, including estimated differences between the means (Estimate), relative standard errors (Std. Error), t- and p-values. Every couple of means is identified here by relative log[Ca^2+^] values:

Simultaneous Tests for General Linear Hypotheses

Multiple Comparisons of Means: Tukey Contrasts

Fit: aov(formula = formula, data = data, weights = weights, x = TRUE)

Linear Hypotheses:

Estimate	Std. Error	t value	Pr(>|t|)

-0.3979 - -0.1549 == 0 0.102600	0.041368 2.4802 0.204521

-0.699 - -0.1549 == 0	0.147000	0.041368 3.5535 0.036119 *

-1 - -0.1549 == 0	-0.033600	0.041368 -0.8122 0.959670

0 - -0.1549 == 0	-0.095200	0.041368 -2.3013 0.265053

1.301 - -0.1549 == 0	-0.062000	0.041368 -1.4987 0.671415

-0.699 - -0.3979 == 0	0.044400	0.041368 1.0733 0.882826

-1 - -0.3979 == 0	-0.136200	0.041368 -3.2924 0.055819 .

0 - -0.3979 == 0	-0.197800	0.041368 -4.7815 0.004540 **

1.301 - -0.3979 == 0	-0.164600	0.041368 -3.9789 0.017532 *

-1 - -0.699 == 0	-0.180600	0.041368 -4.3657 0.009119 **

0 - -0.699 == 0	-0.242200	0.041368 -5.8548 < 0.001 ***

1.301 - -0.699 == 0	-0.209000	0.041368 -5.0522 0.002969 **

0 - -1 == 0	-0.061600	0.041368 -1.4891 0.676889

1.301 - -1 == 0	-0.028400	0.041368 -0.6865 0.980121

1.301 - 0 == 0	0.033200	0.041368 0.8026 0.961613

---

Signif. codes: 0 ‘***’ 0.001 ‘**’ 0.01 ‘*’ 0.05 ‘.’ 0.1 ‘ ’ 1

(Adjusted p values reported -- single-step method)

In order to visualize the experimental values significantly differents, an implemented version of Figure 1 is here reported as Figure S1. The different labels indicate significantly different values.

**Figure S1.** Normalized response of a typical Pt/PPyox/ChO biosensor to PLD 0.22 IU/mL as a function of Ca^2+^ concentration. Experimental conditions as in Figure 2. Different labels indicate significantly different values.

**Figure S2.** Dependence of the choline biosensor sensitivity on the buffer composition (pH 8.5, I 0.1 M). Electrode rotation rate: 1000 rpm.

**Figure S3.** Influence of pH on the response of a typical Pt/PPyox/ChO biosensor. Supporting electrolyte: acetate/phosphate/borate buffer I 0.1 M. Electrode rotation rate: 1000 rpm
